# Universal Free School Breakfast: A Qualitative Process Evaluation According to the Perspectives of Senior Stakeholders

**DOI:** 10.3389/fpubh.2016.00161

**Published:** 2016-08-02

**Authors:** Louise Harvey-Golding, Lynn Margaret Donkin, Margaret Anne Defeyter

**Affiliations:** ^1^Department of Psychology, Faculty of Health and Life Sciences, Northumbria University, Newcastle Upon Tyne, UK; ^2^Directorate of Public Health, Blackpool Council, Blackpool, UK

**Keywords:** school breakfast, school food, child nutrition, food insecurity

## Abstract

In the last decade, the provision of school breakfast has increased significantly in the UK. However, there is an absence of knowledge regarding senior stakeholder views on the processes and potential outcomes on different groups, within the communities served by school breakfast programs. The purpose of this study was to examine the views and experiences of senior level stakeholders and thereby provide an original qualitative contribution to the research. A sample of senior level stakeholders was recruited, including senior officers, directors, and elected members, from within a Local Authority (LA) involved in the leadership, implementation and delivery of a council-wide universal free school breakfast (UFSB) program, and from the senior staff body of mainstream primary and special schools, participating in the program. A grounded theory analysis of the data collected identified issues encountered in the implementation and delivery, and views on the funding and future of a USFB program, in addition to perceived outcomes for children, parents, families, schools, and the wider community. The results refer to both positive and negative issues and implications associated with the program, according to the perspectives of senior level stakeholders. Perceived positive outcomes included benefits to children, families, schools, and the community. For instance, alleviating hunger, improving health outcomes, and conferring financial benefits, with the potential to cumulate in overall improvements in educational, social, and behavioral outcomes. Reported negative implications included the absence of an effective communication strategy in implementing the USFB program; in addition to concerns about the impacts of “double-breakfasting” on obesity levels among children, particularly in less deprived communities. Findings were validated using theoretical sampling and saturation, triangulation methods, member checks, and inter-rater reliability measures. In presenting these findings, this paper provides a unique qualitative insight into the processes, issues and outcomes of a council-wide UFSB program within a socioeconomically deprived community, according to the perceptions of senior level stakeholders.

## Introduction

School breakfast refers to the provision of a breakfast meal for children, usually delivered in schools or alternatively in community and commercial locations. The aims of school breakfast clubs, programs and initiatives are multiple, and may include: alleviating hunger, and improving health and nutrition; alongside the provision of a healthy meal, opportunities for social interaction, educational and physical activities, and out of school childcare. In the UK, school breakfast provision was a relatively recent occurrence during the 1990s, aided by the Government’s Department of Health’s initiative to support the development of school breakfast provision (1). Subsequently, the UK has observed a significant increase in school breakfast programs and clubs in the UK (2), with recent audits indicating that over 85% of UK schools now have some type of breakfast provision (3).

Participation in school breakfast remains relatively low, with accounts of many schools running provision below capacity (3–5). Incidentally, reports of children arriving to school hungry have increased, and breakfast skipping continues to exhibit an increased prevalence among children and adolescents (6, 7). It has been suggested that, while school breakfast provision may hold the potential to mitigate barriers to breakfast consumption among children, more knowledge is required to gain a better understanding of what drives participation and attendance in order develop more effective interventions (8). Universal provision of school breakfast has been suggested as a means of addressing barriers to participation and raising attendance. It is considered that increased participation *via* universal provision may reduce health and educational inequalities and cumulate in wider impacts such as improved punctuality and attendance (5, 9).

It is evident that school breakfast provision originated as a means of reducing health and social inequalities, through the improvement of the nutritional status of children living within areas of high deprivation. However, targeting the neediest children is suggested to establish a stigmatization toward children, families, and communities, which subsequently acts as a barrier to participation (5, 9), with a greater reluctance among children from low income families in utilizing provision when there is an increased risk of being labeled as “poor” (10, 11). Thus, universal provision is considered to reduce the stigma associated with provision for the “neediest” (5). Relatively, USA-based research has reported an increase of 240% in attendance, following the replacement of a means tested school breakfast model for a universally free model (11). Targeting particular groups may be perceived as favorable because resources are directed those who most need them (5, 9). However, it is contested that the reduced costs of targeted provision are negated by increased administrative costs and additional burdens on schools associated with means testing, whereas universal provision removes these inconveniences on schools, in addition to reducing inequalities and stigma (5).

Research highlighting the increased prevalence of breakfast skipping among children and adolescents, particularly those from low socioeconomic backgrounds, older children and females lends further support to universal provision of school breakfast (12–14). Children who consume a breakfast meal are typically considered to have superior nutritional profiles than their peers who skip breakfast (7). Breakfast omission has been associated with increased prevalence of overweight and obesity (12, 15, 16); in addition to a myriad of harmful health behaviors, including consumption of unhealthy fast and snack foods, smoking, alcohol consumption, sedentary behaviors, and weight control behaviors (12, 17, 18). Moreover, research has shown that breakfast skipping may lead to greater declines in cognitive functions, such as attention, across the school day (19). Suggested motives for the omission of breakfast are numerous, and may include socioeconomic factors, such as poverty and food insecurity, in addition to internal factors such as rushed morning routines, fatigue, lack of appetite, and weight control (20).

Qualitative research in the field of school breakfast is limited, and prior research has focused on the sociocultural beliefs, views, and attitudes of children, parents, and school breakfast staff (4, 20–22). Previous qualitative research examining children’s and parents’ views and experiences of school breakfast through interviews, questionnaires, and breakfast club case studies reported that breakfast clubs encouraged breakfast consumption among children, alleviated morning routines, supported parents, and provided childcare (1). A further qualitative study, which examined the views of breakfast club staff recruited from 22 UK schools, found breakfast provision was perceived to be advantageous to children’s health, social development, punctuality and concentration, but also identified concerns about schools appropriating parental responsibility and sustainability of breakfast clubs (23). A US-based focus group study, with parents and children, reported children commonly purchased foods and beverages on the way to school, despite this conflicting with parental rules, and highlighted a need for more communication and collaboration with children and parents regarding school breakfast decisions, in order to raise participation and reduce stigma (4).

There is currently an absence of qualitative research examining the perceptions of senior level stakeholders, involved in the leadership, implementation, and delivery of universal free school breakfast (UFSB). The current study presents findings from a robust and theoretically underpinned qualitative evaluation of the leadership, implementation, delivery, and perceived impacts of a council-wide UFSB program within a deprived town located in the North West of England, UK. The aims of the present study were to examine the views and experiences of senior level stakeholders and thereby provide an original qualitative contribution into the school breakfast phenomenon. A total of nineteen senior level stakeholders participated in semi-structured interviews, including senior officers, directors, and elected members involved in the introduction of the UFSB program, and head teachers and business managers from primary schools participating in the program. The principal research questions included:

1. Did stakeholders experience any issues in the leadership, implementation and delivery of the UFSB program?
2. What were the perceived impacts of the UFSB program on children, parents, families, schools, communities, and the town?

## Materials and Methods

A qualitative grounded theory design was employed to examine the views of senior level stakeholders involved in the implementation and delivery of a LA led USFB program. Qualitative approaches are beneficial in gaining knowledge on governmental interventions by facilitating the exploration of the contexts in which these initiatives operate; highlighting impacts on different groups in the population, and identifying outcomes and factors relating to delivery and organization (24). Purposive and theoretical sampling processes were employed in the recruitment of participants, in accordance with qualitative methodologies, and methods. Data analysis was underpinned by a three stage grounded theory method (open, axial, and selective coding). Validity and credibility of the findings were increased through member checks, use of participant quotations, debriefing participants, and inter-rater reliability. Methods of triangulation were also utilized, including interviews with participants from different fields of the leadership team, and a three staged systematic analysis, which allowed for checking the findings against different data sources and perspectives (25).

Following ethical approval from the Faculty of Health and Life Sciences Ethics Committee at Northumbria University, participants were recruited from the LA, and from the senior staff body of mainstream primary and special schools, participating in the UFSB program. In recruiting participants, a non-probability approach was applied with purposive and theoretical sampling strategies. The purposive element included typical and critical case sampling (26, 27). Participants were recruited on the basis of their position, as pivotal individuals involved in the UFSB program at a senior level. The theoretical sampling approach, allowed for participants to be sampled on the basis of their potential contribution to the development of themes that emerged from the data. Six participants were initially purposively recruited in April 2015, and subsequently a further thirteen participants were recruited using theoretical sampling techniques, over the period of 9 months. The final sample included nineteen participants (Mean = 46 years; range = 32–58), comprising of eight participants from within the LA and eleven participants employed in senior roles within mainstream primary schools or special schools, participating in the UFSB program. All participants were recruited from a town in North West, England, UK. Nine participating schools were located in communities within the bottom half of neighborhoods ranked on indicators of deprivation. The demographic characteristics of each of the nine schools and associated communities are provided in Table 1.

**Table 1:** **School characteristics and school area demographics**.

Schools	School demographics^a^			School and local area demographics^b^		
	Pupils on role ^(N)^	School type	% Pupils entitled to FSM	% All people of working age claiming a key benefit^c^	% White British	Indices of deprivation: total deprivation^d^ (Rank: 1–32,844; 1 = most deprived)
1	398	Voluntary Aided School	12.1	17	96.3	10,346
2	186	Pupil Referral Unit	48.4	17	94.9	9,927
3	53	Community Special School	51.5	16	93.9	13,995
4	194	Voluntary Aided School	14.4	21	96.4	9,270
5	671	Academy Converter	38.5	29	96.41	3,561
6	228	Academy Converter	34.8	44	96.4	188
7	451	Community School	63.2	55	88.0	18
8	450	Voluntary Aided School	13.9	16	95.2	7,600
9	95	Community Special School	54.8	15	95.1	10,779

^a^Information taken from: http://www.education.gov.uk/

^b^Information by ward taken from: http://www.neighbourhood.statistics.gov.uk

*^c^No. of persons, as of March 2010*.

*^d^All 32,844 neighborhoods in England have been ranked on a range of deprivation topics including income, employment, health, education, crime and living environment, with the most deprived neighborhood in England having a rank of 1, as of 2015*.

One-on-one semi-structured interviews were utilized, to allow for a more in-depth exploration of individual perceptions, experiences, and contentious issues (28, 29). Interview schedules comprised of open-ended and probing questions, and as data collection progressed, further probing questions were incorporated, in order to further explore and add detail to emerging concepts and themes (30). A total of 19 interviews were undertaken overall, ranging in timescales between 20 and 90 min. Interviews were audio recorded using a Dictaphone recorder and transcribed verbatim for subsequent analysis. Data collection concluded when it was considered that saturation of categories and themes had been achieved. Theoretical saturation was applied to ascertain saturation, and in generating well developed and cohesive themes and sub-themes, with sufficient variation (30).

**Figure 1:**
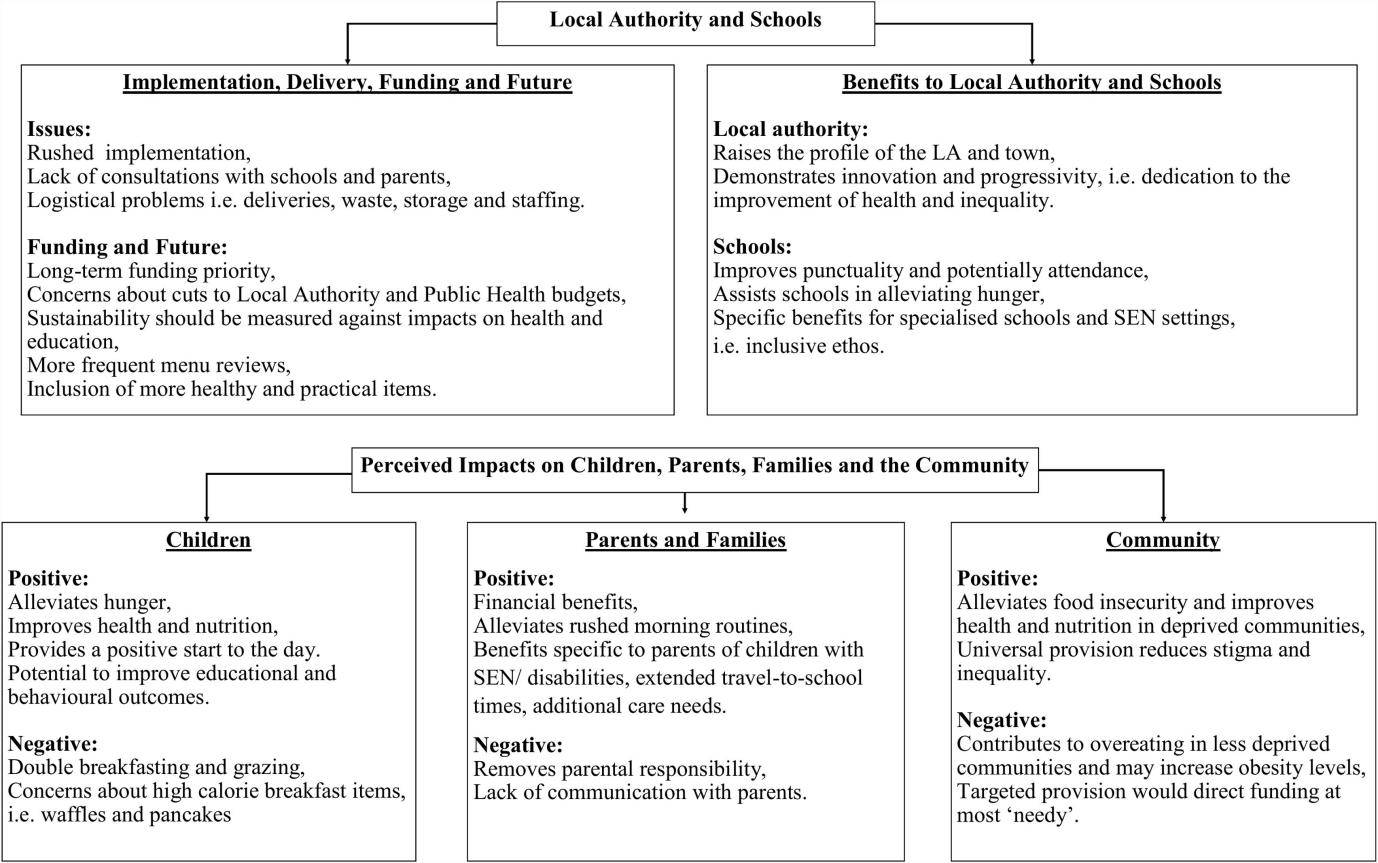
**“Universal free school breakfast: a qualitative process evaluation according to the perspectives of senior stakeholders” – visual model of the findings**.

Data transcripts were the main unit of analysis. Each individual recording was listened to in its entirety and orthographically transcribed to gain a holistic sense of the data. The analysis framework was underpinned by open, axial, and selective coding methods, and constant comparative technique with a focus on conditions, context, and consequences, relating to the phenomenon (31). During the first stage of analysis, all the data were imported into Nvivo for ease of access and organization. Open coding involving a detailed line-by-line reading of the transcripts, adding codes to the data, and writing memos about the theoretical and conceptual ideas that emerged. The subsequent process, axial coding, involved deconstructing and reassembling the data in new ways and making connections between themes and sub-themes (31, 32). The final stage of analysis, selective coding, involved further refinement of the themes and sub-themes into a central model (30–32). This model (Figure 1) provides a framework for understanding the issues encountered in the leadership, implementation and delivery of a UFSB program, and the perceived impacts at child, family, school, and community levels. Reliability analysis was conducted on 10% of the data in accordance with recommendations set out in the literature (33). The second coder providing confirmation that there was 100% agreement between the audio recordings and the corresponding transcripts. Moreover, there was good agreement (Cohen’s Kappa = 0.86) between first and second coders.

## Results

The results of this study, which are visually represented in Figure 1, consist of two key parts. The section “Local Authority and Schools” presents findings associated with the LA and schools; comprising of themes relating to the implementation, delivery, funding, and future of the UFSB program, and supposed benefits to the LA and participating schools. The section “Children, Parents, Families, and the Community” presents themes relative to the perceived benefits of the program for children, parents, and school staff. Findings from each senior stakeholder group are presented separately under each theme as “Group 1” (LA staff) and “Group 2” (senior level school staff).

### Part 1: Local Authority and Schools

#### Issues in the Implementation and Delivery

##### Group 1 – LA

The implementation period of the UFSB program was considered to be rushed, and communications with schools participating in the program were perceived to be inadequate. Head teachers were informed of the Council’s plans to introduce the program during the period when it was announced in the local press. It was acknowledged, in retrospect, that communicating the program to head teachers, subsequent to it being announced in the media, may have made it difficult for schools to opt-out: *“It had been announced in the press and which school was going to say to parents your children are not going to have the free breakfast.”* Thus, it was deemed that the lack of an effective communication strategy may have contributed to issues with schools in the initial stages: *“You set yourself up for issues and complaints and if you don’t [consult].”* Moving forward it was regarded that communication with schools was an area that required improvement, and it was acknowledged that the LA needed to reengage schools, and work with them to gather data on the program and reduce costs: *“We’ve struggled to get the schools to engage with us on it so there’s no ongoing active interest.”*

Issues encountered with schools in the primary stages of delivery of the UFSB program, included problems with storage of breakfast items, food wastage, and additional staffing. These issues were reported to have been resolved through reorganization of food deliveries, funding for additional rubbish collections and reimbursing schools for additional staff time: *“We have bought bins. We’ve paid for additional bin collections. [* … *] We are paying for staff time to prepare and accept deliveries and distribute it out.”* It was considered that high levels of waste in the early stages of the program were due to costing and delivery at 100%: *“It was a universal offer so obviously we costed and planned delivering at 100%.”* Waste was therefore reduced once knowledge was gained on portion sizes across different age groups: *“It settles down and you start to understand portion control and the difference from reception through to year sixes.”*

##### Group 2 – Senior School Staff

The aforementioned issues concerned with rushed implementation and absence of an appropriate communication strategy were also highlighted by senior school staff: *“There was no dialogue. [* … *] It was a question of well you will implement it and if you don’t implement it then you won’t look very good will you?”* The announcement of the UFSB program in local and national media placed schools in a difficult predicament with parents: *“We felt almost forced into implementing the scheme because it was said that every primary school will be serving this. We thought if we are the only school that says to our parents, we are not going to do it, that makes the school look bad.”* Moreover, the introduction of the program was perceived as a top down approach on schools, as opposed to a collaborative approach with schools: *“The council leaders basically stood at the front of a huge meeting of head teachers and said, ‘This is what is happening, you’re all going to have to offer breakfast and you’re all going to do it like this.’”* Additionally, it was thought that the issues with implementation may have been avoided with adequate planning and collaboration with schools: “*We are the people who actually working in the schools delivering the program. We know the sorts of things that they didn’t think about*.”

Issues reported to have been encountered by schools in the implementation of the UFSB program included problems with storage of school breakfast items and high levels waste. It was reported that storage issues were resolved quickly by renegotiating more frequent deliveries: *“We negotiated with the contractors to come twice a week rather than once a week.”* High levels of waste resulted in foods being used by staff, sent home with children, and taken to local food banks: *“A head teacher colleague of mine, on a Friday, on his way home, was going via the food bank and dropping in food.”* Nevertheless, waste was reduced once schools had knowledge about the quantity and choice of breakfast items that children consumed, and could reduce their orders accordingly: *“In the first instance there was an awful lot of waste, but people now are ordering smartly.”* Moreover, some staff reported reducing waste by changing from individual serving model to a self-serve model, resulting in children only taking the food they wanted to eat and being less likely to partially consume foods: “*They help themselves and if they don’t want it they don’t get it.”*

#### Funding and Future of the USFB Program

##### Group 1 – LA

While the USFB program was a priority, it was acknowledged that the council was preparing for up to 40% cuts to overall budgets, and therefore were unable to forecast the impact of such cuts on public spending: *“All councils have been prepared, have been told to prepare for 40% cuts. I have no idea how 40% cuts will impact on councils.”* It was recognized that the cost of the program would need to be examined at some point to assess its sustainability: *“At some point we are going to have to look at whether it is sustainable.”* Moreover, it was considered that the program should be measured against impacts on factors such as educational attainment, attendance and punctuality: *“If you can actually start to measure the impact on educational attainment and attendance, that’ll be a positive impact, which may then justify carrying on with the spend.”* As a significantly deprived town, it was supposed that a considerable number of families would presently rely on the program for a variety of reasons, including low incomes, food insecurity, and rushed morning routines: *“It would really cause people an inconvenience of you took away now, so I don’t see it stopping in the short term.”* There were concerns that terminating the program would have a detrimental impact on children, families, and schools: *“You can’t play with people’s lives to the point that you switch this on and off.”* Therefore, making cuts to the program or changing it in anyway, such as means testing, was considered to be a problematic political decision: *“It’s embedded now and to try and take it away would be very a difficult contentious political decision.”*

##### Group 2 – Senior School Staff

Senior school staff reported that they would like more frequent reviews of the menu and the inclusion of more practical, but healthy breakfast items: *“I think that the council need to look into healthy options, because I know it’s quick for the fact they can just pick it up and eat it there and then, but we’ve got to look at the wider impact on the children.”* There were concerns raised that children may get menu fatigue and interest in the UFSB program may reduce. It was suggested that children should be included in decision making about breakfast items and menus: *“I think they should involve the children in some of the possible products that they offer and involving them in getting their views.”* Moreover, some senior school staff reported that they would also like to explore healthier options such as cereals and porridge. One school claimed that they had expressed an interest in offering cereals and porridge, but were not being supported by the LA to make the necessary changes to move to a full breakfast service model before the start of the school day: *“We had some conversations with the council, but nothing came of it because it almost seemed like it was too hard.”* Conversely, other senior school staff maintained that they felt supported by the LA to deliver more options such as cereal, porridge and toast, but were discouraged to do so by the additional logistical challenges involved: *“It would mean washing bowls, spillage, just the whole milk and cereal, that kind of thing. It’s just not practical.”*

#### Wider Benefits to the Local Authority and Schools

##### Group 1 – LA

It was considered that publicity of the UFSB program raised the profile of the LA and the town nationally: *“It’s given us a lot of credibility.”* Additionally, coinciding the implementation of the program with the introduction of the School Food Plan was perceived to have promoted a positive representation of the LA, as pioneers in working with schools to improve nutrition and diet: *“The director of the School Food Plan came and visited a number of schools, looked at the program in action, and thought it was absolutely amazing.”* Furthermore, it was reported that members of the leadership team were invited to government and public sector conferences to present information about the program: *“XX and myself spoke in the House of Commons regarding this because it’s had so much publicity around the whole of the country.”* It was also claimed other local authorities sought information about the program: *“It has set us apart in the sense that lots of people want to do it, but they have not been able to find that magic key to doing it.”* Ultimately, it was perceived that the program raised the credibility of the Local Council among local and national government: *“It’s been received really well and I think we’ve been seen as ground breaking.”* It was considered that the unique scale and universality of the program, not provided anywhere else within England, demonstrated that the LA were forward-thinking and intuitive: *“We’re quite at the forefront, in terms of doing this. There’s nowhere else in the country offering anything quite like what we’re doing.”*

##### Group 2 – Senior School Staff

Some senior staff felt that the UFSB program had positive impacts on punctuality, due to the appeal of a free breakfast meal at school: *“It’s also helped with getting children into school. They want to be in now because they know that they are going to get their breakfast*;*” “Children like to be pushing their parents to get them into school because they want the breakfast.”* Moreover, in schools that offered breakfast before and at the start of the formal school day, it was maintained that there had been a definite improvement in punctuality, through the provision of a greater window of time to arrive to school: *“Now they can be five minutes late and still be in school okay.”* This additional window also resulted in less congestion around school: *“It helped us because we’ve not had a load of congestion.”* Furthermore, it was thought that availability of a free school breakfast may also have the potential to positively impact on attendance, particularly for children experiencing food insecurity: *“They know that they are going to get their breakfast, they get their dinners. Where the parents are not feeding them they know that they are going to be fed.”*

In contrast to mainstream primary schools, it was reported that special schools catered for children from a wider age range, with all children being offered a free breakfast meal: *“We offer to all our children and we have got 2 to 19-year-olds.”* It was considered that universal provision of the breakfast program was particularly beneficial in special educational needs (SEN) settings because it integrated well with the positive inclusive ethos in schools and did not segregate children based on proxy measures of income: *“It doesn’t make anybody feel different. Everybody is the same. It has got a very positive ethos in school.”* Moreover, senior staff from special schools reported that the program had been incorporated with the curriculum, across a variety of areas including personal, social, health, and economic education, catering and hospitality, and general communication and social skills: “*We have that as part of our life skills. It’s as though you’re working in a café. Obviously food hygiene, how you would present food and how you would communicate with people.”* Finally, the flexible nature of the program was considered by senior staff from special schools to have been advantageous in the implementation: *“It’s quite flexible for us because the Key Stage 2 all come in and have breakfast straightaway. The old ones come in at different times; it enables us to give them their breakfast a bit later.”*

### Part 2: Children, Parents, Families, and the Community

#### Perceived Outcomes for Children

##### Alleviates Hunger and Provides a Healthier Breakfast

###### Group 1 – LA

The fundamental intention in introducing the UFSB program was to ensure that children were no longer hungry across the school morning. Prior to the introduction of the program it was claimed that children were arriving to school hungry, some having not eaten since their last meal at school: *“The information we were getting back from schools around the number of kids who were coming in having not eaten and teachers providing food.”* Formerly, provision for hungry children was reported to have been met by individual schools and teachers, on an *ad hoc* basis: *“Individual teachers, individual welfare assistants and learning support assistants, actually paying for some kind of food out of their own money.”* This was deemed unacceptable by the LA; hence the introduction of the program, and ensuring that all primary school children had the opportunity of a breakfast meal: *“That made quite clearly that there was a definite need first thing in the day.”* However, it was considered that while the town overall experienced high levels of deprivation, there was a greater need for the program among the more deprived communities within the town: *“The scheme is being more formally welcomed in the very deprived areas. They were the ones that had to deal with the kids that were arriving malnourished and that’s inevitable.”*

It was also thought that school breakfast may be typically healthier than breakfast at home or on the way to school: *“They may not be getting a healthy breakfast at home. If we can promote a healthy breakfast in school then that benefits them [children].”* It was perceived that school breakfast perhaps provided some children with a wider choice than they may encounter at home. Additionally, it was felt that school breakfast provided children with autonomy and independence to make their own decisions and choices, and perhaps encouraged children to try new foods: *“I would hope that children were trying things that they didn’t try previously.”* Moreover, it was believed that eating with their peers may encourage children to sample different foods: *“Peer pressure is a great thing, so if their friend’s trying something they tend to try it.”* Since the introduction of the UFSB program, it was thought that children were less likely to buy high calorie foods and beverages to consume on the way to school, and parents were also less likely to give children money to buy breakfast on the way to school: *“Parents are less likely to give them money as well. [* … *] They can say well get your breakfast when you get to school.”*

###### Group 2 – Senior School Staff

Senior school staff also considered that the UFSB program alleviated hunger among children in schools: *“It ensures that all our young people have the opportunity of having something to eat first thing in the morning. I know some of our young people are very hungry. The fact that the food is always there is reassuring for them.”* It was also thought that the program bridged the gap between breakfast and lunch for children who received an early breakfast, perhaps due to parents leaving early for work or travel to school times: *“The youngster who has already had porridge at 6:30 that morning, you’re looking at two and half hours later. It’s a long time to lunchtime, so it bridges that gap.”* Moreover, feeding hungry children in the mornings was believed to be easier for schools since the introduction of the program because it afforded easy access to practical food items: *“If they do say that they’re hungry, I’ve got the resource there. I don’t need to go to breakfast club stores and pull it out of my reserves. It’s there and it’s available.”*

School staff also supposed that for some children, the breakfast meal served at school may be healthier than breakfast at home or on the way to school: *“We’ve got children seeing healthy breakfast displayed in the morning. It’s fresh and it’s good for them”* School breakfast was thought to provide children with independence to make choices about the foods they wanted to eat and potentially encourage children to try new foods that they may not have previously consumed, *“It’s actually given them the choice. You’ve taken away, you will eat this or you won’t eat it.”* Prior to the introduction of the UFSB program, staff reported that some children arrived to school in the mornings with high calorie/low nutrition snack foods and beverages, such as crisps, and carbonated drinks: *“They would often come into school and buy a can of pop on the way, crisps on the way and eat that for breakfast.”* It was deemed that these behaviors had reduced since the introduction of the program: *“I have not seen in months, children walking to school eating junk. I did see that before breakfast came along.”* Additionally, it was considered that parents were less likely to provide children with money to buy breakfast on the way to school since the introduction of the program: *“Some of our parents here would give their kids money to get breakfast on the way to school, so they might get a bag of crisps or bags of sweets. Now they are coming into school and getting something of some nutritional value.”*

##### Perceived Educational Benefits

###### Group 1 – LA

In addition to alleviating hunger, raising educational attainment was also an aim of the UFSB program. Feeding hungry children in the morning was considered a means of improving attainment, by ensuring children were not hungry and thereby ready for learning: *“We have poor levels of educational attainment. It’s great that we can feed those kids properly, so that they are ready to learn.”* With regards to impacts on educational outcomes other than learning, it was considered that program had the potential to improve punctuality because children were more like to arrive on time: *“I am aware that kids now get to school earlier than what they did.”* Moreover, for children from chaotic or working households, where time may be limited in the morning, it was perceived that the program provided children with a calm and positive start to the day. This positive start to the day was also presumed to provide children with social development opportunities to interact with their peers and staff while eating breakfast, which was thought may not be feasible every morning at home: *“The fact the children sit down with their friends and have their breakfast. I do believe that it has a very calming influence.”* This was deemed to have beneficial impacts on the school environment and classroom behavior: *“The key feedback from heads is that they are ready to learn and breakfast plays an important role in it.”* It was supposed that by alleviating hunger, and improving the school environment, free school breakfast would contributed to happier, healthier, well-nourished and attentive children, leading to longer term impacts on educational attainment.

###### Group 2 – Senior School Staff

It was considered among staff that school breakfast reduced children’s anxieties about being hungry and therefore was beneficial for learning: *“It reduces their anxiety and their anger because they know that they are going to get something when they get to school*;” “*It improves their behavior because they are not hungry.”* It was apparent that since the introduction of the UFSB program, children were no longer losing concentration in class due to hunger: *“We don’t have hungry children who lose focus and attention.”* Likewise, school breakfast was also considered to impact positively on the school environment, providing a calm and positive start to the school day: *“They can have a bit of time and a bit of something to eat with their friends, and maybe read a book. It is quite a nice time in the classroom that is a positive experience for them.”* Furthermore, it was considered that school breakfast provided schools resources to feed children quickly, meaning that staff were spending less time on feeding hungry children and more time on facilitating learning: *“The fact that the breakfast that we’ve got is easily available and you can just give them something quite quickly. It means that once they get into lessons they can start learning.”* In the longer term, it was envisaged these positive outcomes on learning, behavior and the school environment, would have a positive impact on educational attainment.

##### Double-Breakfasting and Grazing across the Morning

###### Group 1 – LA

LA staff were aware of concerns about the potential detrimental impacts of the UFSB program on children, such as encouraging overeating and increasing obesity levels: *“There is some concern that maybe some children are double eating in the morning.”* It was perceived that a small minority of children, perhaps from more affluent areas of the community, may be eating breakfast at home and at school: *“If they are getting an unhealthy breakfast at home in the more affluent families, and then getting a healthy one at school it is still adding calories.”* However, it was contested that concerns about children overeating in the mornings may be exaggerated and conceivably most children would not continue to consume foods and beverages once they were satiated: *“If they are not particularly hungry, if they only want a piece of fruit in the morning that is all they will take. They don’t cram it in.”* Moreover, it was highlighted that some children may be receiving an early breakfast, due to having working parents or a longer travel time to school, and may therefore be hungry during the school morning. It was perceived that school breakfast ensured that all hungry children were provided for regardless of the circumstances. In order to prevent potentially undesirable impacts of double-breakfasting, it was considered that communications with parents needed to be improved as a matter of urgency: *“We need to get the right message to parents that we will be providing breakfast for those kids.”*

###### Group 2 – Senior School Staff

There were concerns raised about the potential impact of free school breakfast on obesity levels, due to increased opportunities for grazing and “double-breakfasting” behaviors. It was perceived that in some instances school breakfast may be being treated as a mid-morning snack: *“For some children it’s just a complimentary snack,”* which was felt by some to contravene the fundamental purpose of the UFSB program. There were concerns raised that some children may be eating more than one breakfast across different locations in the morning, including at home, on the way to school and at school *“So they have breakfast at home, breakfast at breakfast club, and then they come into school and have bread product.”* Moreover, it was considered that a number of parents were still providing children with a breakfast, irrespective of the free breakfast served at school: *“A lot of parents said to me privately that, ‘We don’t need the scheme. I feed my child in the morning. I believe it’s my responsibility to feed my children.’”* There were apprehensions that these children may consequently be overeating across the morning, which may lead to detrimental impacts on obesity levels: *“There may well be some negative features of feeding children who don’t need a second breakfast.”* In one school, it was considered that the program contravened healthy diet initiatives and encouraged children to eat more regularly: *“We are doing all sorts of healthy eating work, we are using the pupil premium money to try and get children fitter and healthier. [* … *] Then we are chucking all the sugar and stodge at them in the morning.”* Notably, it was believed that the likelihood of “double-breakfasting”’ was more probable when free school breakfast was served in the classroom, where food was available to all children: “*They just have it anyway because it’s there, because children will eat if there is food there.”*

Reported strategies for managing “double-breakfasting” in schools included discreetly managing portions for children who had already eaten breakfast: *“We do have to try and get around it and we do that by cutting the toast into smaller pieces, offering half a crumpet.”* Additionally, communications with parents were thought to be particularly important in managing “double breakfasting,” especially in SEN settings, where children may have care, diet, and health plans in place as part of safeguarding their wellbeing: *“Through discussing with the parents we have managed to reach individual conclusions and solutions to that problem. All of our children have care plans and diet plans, so it is done on a very individual basis.”* Furthermore, some senior school staff contended that generally most children were capable of managing their food consumption across the morning and were unlikely to overeat free school breakfast items: *“Children don’t overeat. They are not just going to sit there and eat and eat.”* Furthermore, some senior staff highlighted that children who receive an early breakfast may be hungry by mid-morning, and therefore may require an additional snack to satiate them until lunch time: *“Some of our children might have their breakfast at 7:30, but then it’s five hours before they have their lunch. We are quite careful about what they do have.”*

##### High Sugar/Unhealthy School Breakfast Items

###### Group 1 – LA

Further issues raised were problems encountered with high-sugar school breakfast items and concerns about obesity and dental health among children. In the initial stages of the UFSB program, fruit juice and sugary breakfast bars were available on the school breakfast menu, but due to concerns about sugar content, these items were removed: *“We used have fruit juice on. That’s now had to come off. [* … *] It was public health, because it has an impact on dental health.”* It was highlighted that the nutritional value of the school breakfast menu had to be balanced against providing items that children will eat, and items that were practical in the context of the different environments they were served: *“You’ve got to go with something that is fairly easy to eat with your hands, that doesn’t require a lot of clearing up afterwards.”* Moreover, it was reported that providing practical breakfast items that were also healthy in terms of balanced nutrition was also difficult: *“The handheld one does offer a real challenge in getting a good variety of healthy items in there.”* Some staff thought that serving children foods such as cereals, porridge, and hot foods may be healthier for children. However, it was acknowledged that the delivery of these items in different school environments was challenging and not feasible for some schools, *“You’re not able to serve porridge or hot food very easily or toast.”* Moving forward, it was considered that further discussions were required with suppliers to improve menu choices and explore the provision of cereal and porridge.

###### Group 2 – Senior School Staff

There were concerns raised that the food items served to children, including ready-made pancakes, waffles and brioche, were too high in sugars and fat: *“I was looking at one of the waffles, I’ve got the pack in front of me here and the, on the traffic light system they have now on the packets, it’s red for fat, saturates and sugars. Each waffle contains 93 calories and, 5.2 g of fat.”* It was deemed that the provision of these items should be reviewed by the LA to assess the impact on dietary and dental health: *“Apart from leading to obesity there’s the impact on the teeth as well. It’s not probably encouraging very good eating habits, having very sweet items available every single morning.”* It was contended that the provision of these particular foods as mid-morning snacks, potentially fostered detrimental eating habits, which may impact on dental health and obesity: *“You wonder why you have got real problems with tooth decay. We are certainly not teaching a good pattern of eating by the time we have breakfast so close to the lunch.”* Furthermore, it was asserted, by some senior staff, that the provision high calorie, hand-held items, for free school breakfast, also contributed to harmful dietary behaviors associated with obesity: *“Perpetuating fast-food mentality. It’s just reinforcing the McDonald’s mentality of something quick and easy and it’s full of sugar.”*

#### Perceived Outcomes for Parents and Families

##### Financial Benefits for Parents and Families

###### Group 1 – LA

It was considered that the UFSB program provided a small level of financial assistance to parents and families: *“Parents that would otherwise struggle, are still struggling, but hopefully it will just make things a little bit easier for them.”* This was considered to be particularly beneficial for those families on low incomes, who may be experiencing poverty and food insecurity, and those parents with multiple children: *“I think it’s an amazing scheme if you have got one, two or three kids in particular.”* Additionally, it was also supposed that the program conferred financial benefits for parents who previously provided children with money to purchase breakfast on the way to school. Moreover, it was thought that providing this small level of financial assistance to parents and families may also lead to wider impacts on the local economy, with money being directed into other areas, such as the evening meal: *“That must release some of your money that you’ve got to be able to spend in other ways and hopefully redirect it to food in the evening.”*

###### Group 2 – Senior School Staff

Likewise, it was also considered among senior school staff that school breakfast provided parents with a small level of financial assistance, which was thought may be especially significant to those on low incomes and families with multiple children. Furthermore, the UFSB program was supposed to be of benefit to parents who previously provided children with money to purchase breakfast on the way to school: *“The parents I know who are maybe a little bit financially better off because they’re not having to give the kids money to give them something on the way to school.”* Moreover, universal provision of school breakfast was also perceived to offer a small level of financial assistance to those parents not eligible for free school meals, but still be on low incomes: *“I’ve also got some parents who are just on, kind of the borderline, just slightly too much to be in receipt of pupil premium. A breakfast for their child every day is saving a little bit of money for them which they can then spend in different ways.”*

##### Alleviates Rushed Morning Routines for Families

###### Group 1 – LA

The UFSB program was also thought to alleviate rushed morning routines for families. It was deemed that the provision of a breakfast meal at school reduced stress for families during school mornings: *“It is a very difficult time of the day and we all get it wrong sometimes. We all run out of time to do hair, to sort out shoes, school bags, and no matter how much you try the night before, it’s difficult.”* Factors assumed to contribute to the school morning rush for parents included work and educational commitments, household responsibilities, and caring for very young children and/or multiple children: *“It’s about households being very busy. If there are five children in the house, mum and dad both work shifts. Everyone’s busy and everyone’s hurrying about.”* Additionally, it was thought that modern lifestyles resulted in the majority of families not having time to sit together and eat breakfast on a school morning: *“The number of families that do actually do a full sit down breakfast altogether is limited.”* As opposed to this social time in the morning being eroded, since the introduction of the UFSB program, it was considered children had gained social interaction with their peers at breakfast time.

###### Group 2 – Senior School Staff

The UFSB program was also considered by senior school staff to alleviate rushed morning routines for families for a variety of reasons. *“I think it’s just taken that little bit of pressure. If they oversleep they don’t have to say, ‘Oh God we haven’t had a breakfast, I’ve got this to do, I’ve got that to do and then I’ve got the house to clean:’” “I have got lots of professional parents who in the morning don’t have time to give their children breakfast. Full-time working parents need to be at work.”* Moreover, it was highlighted that families with very young and/or multiple children may experience additional pressures in the morning: *“I have parents who, their children come to school on their own because they’ve got other drop-offs, they might have younger children or older children.”* It was supposed that a free school breakfast helped to alleviate rushed mornings by saving time spent on making and eating breakfast for children and assisting parents in getting children to school on time: *“Mornings can be very busy, especially if you’ve got a lot of children, so it might be something that means they’re going to get to school on time.”*

##### Benefits Specific to Parents of Children with SEN/Disabilities

###### Group 2 – Senior School Staff

It was considered that free school breakfast provided support to parents of children with SEN and/or disabilities. It was described that morning routines for these families may be more complicated, particularly for parents of children with profound and/or complex SEN/disabilities, for whom everyday tasks required additional care, assistance and time: “*Most of them need support for dressing, support to get into a wheelchair, support feeding. Kids aren’t able to get out of bed at the last minute and get up and down to transport.”* Moreover, it was reported that children attending SEN schools, often had further to travel to school and/or were provided with transport, which often meant leaving home earlier in the morning: *“All of our children are bussed into school. They are collected from home because they are actually needing to be transported to school.”* In these instances, it was perceived that UFSB program was advantageous to children and their families, by both alleviating morning routines and providing children with a breakfast subsequent to a potentially very early morning and longer journey to school: *“They might have been up since 6:30 and arriving at school quarter to nine, so already they have had quite a significant morning, in the respect of getting out of bed and getting ready, so they are ready for something.”*

##### Removes Parental Responsibility

###### Group 1 – LA

It was acknowledged that there were concerns raised in the community about the UFSB program removing parental responsibility and facilitating detrimental parenting behaviors: “*The only potential negative really is on that dependency issue. There are a number of different elements of children’s lives that unfortunately sometimes parents think I don’t have to do that.”* Nevertheless, it was deemed that as opposed to fostering poor parenting traits, the introduction of the program showed recognition that children were arriving to school hungry, and aimed to feed these children, irrespective of their parents’ behaviors. It was also contended, that within an area of such significant levels of deprivation, experiencing high levels of alcohol and substance abuse, domestic violence and neglect, there would inherently be parents with chaotic lifestyles: *“Neglect is our biggest issue. There are a lot of stresses and strains, drug and alcohol abuse, domestic violence. There may be lots of chaos, and we need to lift up some of these burdens.”* The program was thought to facilitate families in getting children to school on time, in addition to providing children with a breakfast meal and positive start to the day.

###### Group 2 – Senior School Staff

It was perceived that the UFSB program may remove parental responsibility and promote poor parenting behaviors: *“What you are doing is you are saving parents the hassle and deskilling them.”* Concerns were raised about removing parents’ responsibility to provide their children with a breakfast meal, and placing that responsibility on the school and the state. There were apprehensions that this may deskill parents and create a generation of families who do not eat breakfast together at home. However, some school staff contended that within an area of such high deprivation, it was inherent that some parents would send their children to school without breakfast: *“You tend to think that everybody is like yourself and you would send your child off to school having had a good breakfast but that isn’t the case.”* As opposed to deskilling parents, some senior school staff maintained that the UFSB program demonstrated recognition of the reality for some children living in the community and experiencing deprivation: *“It’s that understanding of what life could be like for some of our children. It’s not these kids’ faults that they have not got parents who are able to provide breakfast.”*

##### Lack of Communication with Parents

###### Group 1 – LA

It was presumed that schools were in a better position to communicate the UFSB program to parents due to already having an existing a relationship: *“Those parents have more of a relationship with teachers and schools.”* It was deemed that centralized communication of the program to parents *via* the LA would be difficult due to the variation in models across different schools: *“To try and communicate 20 different models of what’s happening. We can’t say to every parent, ‘your child can arrive at school 8.30 – 8:45 and they will have a breakfast comparable to what you would have at home.’”* Communication with parents was an area that was deemed to require improvement, in order to reduce the likelihood of the potential detrimental effects, such as double-breakfasting and contribution to obesity: *“There’s more we need to do in relation to parents understanding this.”* It was considered that parents should be provided with the appropriate knowledge about the breakfast items served as part of the program in order to make informed decisions about providing breakfast at home.

###### Group 2 – Senior School Staff

It was reported that in the initial stages of the UFSB program, there were criticisms from parents, who were offended by the perception that parents in the town were unable to feed their children a breakfast meal. Some parents had allegedly complained to schools that they were able to provide their children with breakfast and did not require the program: *“A lot of parents were quite shocked at the fact that we were saying they weren’t feeding their children. [* … *] Some parents thought that it was patronizing; that they were being criticized about their parenting.”* It was supposed that these negative parental perceptions may have been overcome through more effective communication with parents: *“I think if they took a little bit more time to go out to parents that would have helped.”* It was deemed that communication with parents required improvement, in order to identify parents’ views about the program, and involve them in the decision making processes: *“I think they need to be aware of what we are providing and I think we need to get their opinions on what is best for their children.”* It was also judged that better communication was necessitated with parents to prevent issues such as overeating and “double breakfasting” behaviors: *“I think they should do more surveys saying what is it your child wants, does your child have breakfast.”*

#### Perceived Impacts at a Community Level

##### Outcomes in Communities with Different Levels of Deprivation

###### Group 1 – LA

The community served by the UFSB program experiences significant and multiple levels of deprivation, including high levels of child poverty, poor dietary health, obesity issues, and poor educational attainment: *“Child poverty rates, our poor diet and nutrition issues, obesity levels, poor educational attainment.”* It was reported that prior to the introduction of the program, individual schools were thought to be taking measures to alleviate food insecurity by feeding hungry children, providing breakfast tokens outside of the school term, sign posting to food banks, and through small weekend money lending schemes. It was asserted that the UFSB program helped to mitigate food insecurity and health inequalities associated with high levels of deprivation: *“They are born into diabetes, being overweight, poverty, poor housing. As things stand their life expectancy is one of the worst in the UK.”* It was acknowledged that the program perhaps had a greater impact on the more deprived communities: *“It’s been very supportive in terms of families in deprived areas where they haven’t got the resources to provide a nutritious breakfast.”*

###### Group 2 – Senior School Staff

Likewise, it was considered that the UFSB program had different impacts in different communities within the town. Senior staff, in less deprived wards, with lower levels of children eligible for free school meals, considered there was probably less of a need for a universal program within their schools: “*There isn’t necessarily a need for all of ours. We have got something like 8 of our 400 children who are pupil premium and that tends to be free school meals.”* There were concerns that within schools in less deprived areas more children may be “double-breakfasting,” and the UFSB program may be having longer term detrimental impacts on obesity: *“It’s just a case of children eating food that they don’t really need. Hence, maybe children will actually become overweight.”* However in the more deprived communities, the program was thought to be extremely beneficial to families experiencing poverty and food insecurity: *“The council has recognized the deprivation. Whether it’s down to a parenting skill or whether it’s down to not having enough money, it’s a way of getting to the root of the issue, which is making sure that the children get fed.”* It was perceived that the program was fundamental in these communities where there were extremely high levels of deprivation and more children were arriving to school hungry: *“Many of them don’t have breakfast in the morning, so providing a small breakfast for them is one positive way that we can reduce the barriers that some of our children have.”*

#### Universal vs Targeted Provision

##### Group 1 – LA

The universality of the UFSB program was considered to be an important factor in increasing participation and reducing social stigma: *“We have a universal scheme and that’s the great thing that all our kids get this, so there’s no stigmatization of the poor kids.”* It was reported that the LA had received criticism from more affluent communities and the political opposition for delivering the program on a universal basis, as opposed to potentially more economical means tested program targeting the most needy families and children: *“There has been a political argument within the council around the opposition saying should it be means tested and that it would reduce the cost.”* Nonetheless, it was disputed that savings in costs through means testing would be negated by increased administration and burdens on schools: “*With means testing you end up spending more on the admin.”* Additionally, it was contended that means testing would result in stigmatization of the program and have detrimental impacts on participation: *“I think it would cause more problems and stigmatize things.”* Furthermore, it was argued that the high levels of participation in the program demonstrated that it was utilized by families at both ends of the income scale: *“It is appreciated, by not just those at the bottom end of the income scale, it’s taken up by those with slightly higher incomes.”* It was also noted that the average income in the town was relatively low on national comparisons, which was also considered to be relative to the case for universal provision: *“The average wage is fairly low here anyway. There aren’t many families out there who are earning so much money that they’d be able to pass up the possibility of a free meal.”* Ultimately, the universal element of the program was considered to be a key factor in embedding it within the community and creating a positive ethos: *“The universality of it is a really important feature in terms of reducing inequalities in the town, and not stigmatizing it in any shape, way or form. It is a program that is for everybody and everybody can benefit from it.”*

##### Group 2 – Senior School Staff

It was considered that universal provision of the UFSB program was necessary in order to remove barriers, maintain equality, and eliminate stigma. It was deemed that it would be extremely difficult for schools to provide breakfast to a targeted group of children, especially younger children or those with SEN: “*If it was means tested we couldn’t say to our children you can have it but you can’t.”* Furthermore, universal provision was considered to be extremely important in SEN schools, where the universal element of the program was aligned with an inclusive ethos: *“It doesn’t make anybody feel different. Everybody is the same. It has got a very positive ethos in school.”* Conversely, other senior staff from less deprived wards felt that a more targeted approach would have had a greater impact at a lower cost: *“I mean it’s very expensive and I’d like to think it was more targeted, rather than universal.”* Potential suggestions for a means tested program included schools using their knowledge of families and children to offer targeted support: *“It should be done on our knowledge of the family.”* This approach was favored as opposed to using measures such as free school meals: *“Whenever you do means testing, it always comes back to free school dinners and that’s wrong because we have a lot of deprived children who have working parents on minimum wage, who are seasonal workers.”*

## Discussion

According to the perspectives of the senior level stakeholders, the outcomes and issues associated with a council-wide UFSB program were multiple and varied, and both positive and negative. A recurrent issue was the absence of an effective communication strategy, primarily related to a lack of appropriate and timely communication by the LA, to schools, parents, and the wider community. The implementation process of the UFSB program was perceived to be impetuous, and did not allow for appropriate consultations with other stakeholders and hence contributed to some of the perceived negative outcomes of the program. School initiatives to improve health behaviors have been shown to be more effective when a collaborative approach is employed with the cooperation of various sectors of the community, including families, third sector organizations, government, and the media (34, 35). It is apparent that the success of school level healthy eating interventions, such as UFSB programs, to some extent depends on the effectiveness of the communication strategies between key stakeholders. Thus, communication is an area, which requires careful consideration when implementing such programs.

In spite of the aforementioned issues with communication, the program was associated with a range of perceived positive outcomes for children, parents, families, schools, and the wider community. For children, positive outcomes included alleviating hunger, and improving diet and nutrition; thereby cumulating in improved educational, behavioral and social outcomes. It was agreed among senior stakeholders that the UFSB program achieved its fundamental aim of alleviating hunger during the school morning. However, it was contended that the impact was greater in schools located in more deprived communities. Research suggests that breakfast omission behaviors have an increased prevalence among children from lower socioeconomic backgrounds, which consequently contributes to health inequalities within deprived communities (9, 36). Universal provision is considered to play an important role in reducing health inequalities in deprived communities by facilitating healthy breakfast behaviors and removing barriers to participation (37). Therefore, while in this study, there were concerns about reverse negative impacts on less deprived areas of the community, it is evident that the perceived disproportionate advantages of the program in more deprived schools may reduce health inequalities in the wider community.

Despite the consensus that the program alleviated morning hunger in primary school children, there were mixed views regarding the nutritional value of some the food items served. Concerns pertained to high levels of sugars and fats within brioche, waffle and pancake items, and the potential contribution to increased obesity levels and declines in dental health, among children. It has been contended that UFSB programs enhance nutritional intake among children from low income families, and programs have typically been employed in the US as interventions to alleviate hunger and improve nutritional deficiencies (38). Moreover, research has demonstrated that access to school breakfast can considerably improve the nutritional quality of children’s diets (39), particularly for children from deprived households, and thereby reduce health inequalities (40). A practical solution in mitigating the issue of serving high calorie hand-held school breakfast items, would be for school breakfast programs to explore more cereal based options, although the challenges in providing cereals is acknowledged in the findings of this current study. Notably, research has shown that children and adolescents who consume breakfast cereals regularly have significantly lower BMI levels, and it has been suggested that regular consumption of breakfast cereals results in a reduced likelihood of being overweight/obese (41).

In addition to concerns regarding the nutritional value of some of the food items served, there were also apprehensions raised regarding the potential for children to overeat across the school morning. A recent study into a UK school breakfast program reported that almost half of participating children had eaten breakfast items at home and at school; however, an analysis of total energy showed no significant differences between children who ate two breakfasts and those who ate one (40). Research, into the USDA’s School Breakfast Program, reported that the availability of UFSB was associated with an increased likelihood of eating more than one breakfast at home and school (42). Those among the cohort who consumed an additional breakfast had higher energy intakes at breakfast and across 24 h, but notably fat and sugar intakes across 24 h did not differ significantly between groups (42). Interestingly, longitudinal research has shown significant increased odds of overweight/obesity among breakfast skippers, compared to double breakfast eaters, with double breakfast eaters being likely to be within a healthy weight range (6). The evidence suggesting that those students who eat two breakfasts are not significantly more likely to be overweight/obese appears paradoxical, because ultimately an additional meal is being consumed. It has been suggested that perhaps double breakfast eaters may be more physically active and expand more energy throughout the day, or that eating a higher number of calories in the morning is compensated by reduced calories consumption later in the day (6). Additional research is required to examine energy intake across a whole day for those who skip breakfast, consume one breakfast, or consume more than one breakfast, in order to gain a more comprehensive understanding of the associations between breakfast, calorie intake, and adiposity, in children and adolescents.

Finally, with regards to parents and families, UFSB was considered to confer financial benefits, alleviate food insecurity, and reduce stress associated with rushed morning routines. Previous research has highlighted that school breakfast alleviates morning routines for parents with employment and/or caring responsibilities, particularly where school breakfast is served earlier before the start of the school day, thereby providing an element of child care (20, 43). Moreover, school breakfast provision is considered to ease rushed mornings for families who face lengthy journeys to school and/or work, or live in chaotic households (20, 43). It has also been suggested that free school breakfast provision is beneficial to families, especially those experiencing food insecurity, providing a small financial benefit to parents (43, 44).

However, in spite of these supposed benefits for parents, in this paper, there were also concerns raised among senior stakeholders that UFSB encourages detrimental parenting behaviors, by removing parental responsibility to provide breakfast, leading to further dependency, and an erosion of traditional family meal times. It has been proposed that consumption of breakfast at home may be an indicator of a child’s home environment and the capacity for parents/carers to provide a nurturing environment for children (45). However, it has been contested that the presumption that a child’s breakfast consumption at home is a predictor of “good parenting,” is not currently grounded in scientific evidence, with many well-intentioned child feeding practices, such as pressuring and restricting, being ineffectual and potentially counterproductive (9, 45). It is asserted that prevalent levels of breakfast skipping among children and adolescents, cannot be solely explained by parental failures in providing a nurturing environment, and while parenting is evidently an important socio-ecological feature affecting how young people eat, it is not the sole influencing factor (45).

The current study offers an original insight into the phenomenon of school breakfast provision and expands on current qualitative research in the field with parents, children, and school staff. The results of the present study lend support to previous qualitative research highlighting reports that school breakfast provision encourages breakfast consumption, alleviates morning routines for families, and could be advantageous in improving children’s health, social development, and educational outcomes (1, 20, 23). The findings also support prior qualitative research in the US, highlighting the importance of effective communication strategies with parents and children (4). However, while this paper provides a unique and insightful contribution into the leadership, management, implementation, delivery, and perceived benefits of a UFSB program, from the perspectives of senior level stakeholders, the study is not without limitations. The ability to generalize the findings from this study is restricted, due to the research being carried out within a highly deprived council within the North West of England, UK, and not necessary representative of the UK population. However, considering that school breakfast interventions are often targeted at deprived communities, the findings from the current study may have particular relevance as a point of reference to those involved in the leadership, management, implementation, and/or delivery of school breakfast programs. It should be noted that the aim of this study was not to infer causality or wide ranging generalization, but instead to present an account seniors stakeholders’ experiences and perceptions of the leadership, implementation and delivery a UFSB program and supposed outcomes at individual, school, family, and community levels. Further research is necessitated, particularly at a national level in the UK, in order to gain a more comprehensive knowledge into the effectiveness of school breakfast, in the context of outcomes relating to the child, school, family, and wider community.

## Author Contributions

All authors made substantial contributions research design, the recruitment of participants, the acquisition of data, and the analysis of the data. In addition, all authors participated in drafting and critically revising the manuscript. Finally, all authors have approved the final submission and agree that they are all accountable for all aspects of the research and final work.

## Conflict of Interest Statement

The authors declare that the research was conducted in the absence of any commercial or financial relationships that could be construed as a potential conflict of interest.
